# Cerebral Hypoperfusion Caused by Brachiocephalic Artery Stenosis

**DOI:** 10.1016/j.ejvsvf.2025.09.005

**Published:** 2025-09-17

**Authors:** Nusr Ghamri, Donald Harris, David Lindström, Anastasia Dean

**Affiliations:** a Faculty of Medical and Health Sciences, University of Auckland, Auckland, New Zealand; b Auckland Regional Vascular Service, Auckland City Hospital, Auckland, New Zealand; c Section of Vascular Surgery, Department of Clinical Science and Education, Karolinska Institutet, Södersjukhuset, Stockholm, Sweden

**Keywords:** Brachiocephalic artery disease, Case report, Cerebral hypoperfusion, Endovascular revascularisation, Brachiocephalic artery, Transient ischaemic attacks

## Abstract

**Introduction:**

Extracranial cerebrovascular disease can cause cerebral ischaemia through embolism or hypoperfusion. Managing cerebral ischaemia in patients with hypoperfusion and multivessel cerebrovascular disease can pose challenges owing to the risks of embolisation and haemodynamic instability, especially when normal embolisation protection techniques and cross clamping are hazardous.

**Report:**

This article presents the case of a 74 year old woman who experienced a peri-operative cardiac arrest during femoropopliteal bypass surgery, secondary to undiagnosed severe left ventricular hypertrophy with dynamic outflow obstruction. Following recovery, she developed recurrent right hemispheric transient ischaemic attacks including left hemiplegia. Imaging revealed mild to moderate bilateral carotid bulb, carotid siphon, and vertebral stenoses, but the most significant lesion was a severe, calcified stenosis of brachiocephalic artery. Given the recent cardiac arrest and multiple levels of the disease, the initial plan was for conservative management. Despite medical management with permissive hypertension, the patient continued to experience transient ischaemic attacks as soon as the systolic pressure dropped below 160 mmHg. This scenario led to a multidisciplinary decision to proceed with brachiocephalic artery stenting. The neurointerventional team recommended avoidance of cross clamping if possible given the severe lesions and lack of intact circle of Willis. The procedure was done under general anaesthesia via open, retrograde right axillary access without carotid cross clamping. The post-operative course was uneventful.

**Discussion:**

This case underscores the importance of procedural planning and a multidisciplinary approach in managing complex cerebrovascular conditions, and that unusual pathologies may need unusual treatment.

## Highlights

• A case of retrograde innominate artery stenting to treat recurrent TIAs due to hypoperfusion.
• Multidisciplinary collaboration facilitated a tailored, minimally invasive approach for a high risk patient.
• Stenting resolved cerebral hypoperfusion with no recurrence of neurological symptoms post-operatively.

## INTRODUCTION

**Figure 1:**
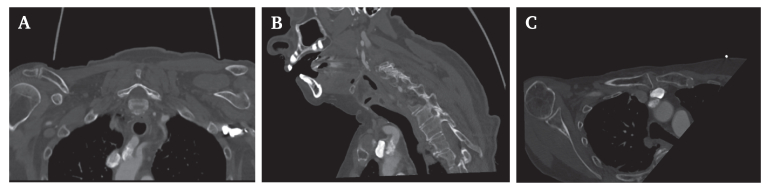
Computed tomography scan showing brachiocephalic artery lesion stenosis. (A) Coronal. (B) Sagittal. (C) Axial.

**Figure 2:**
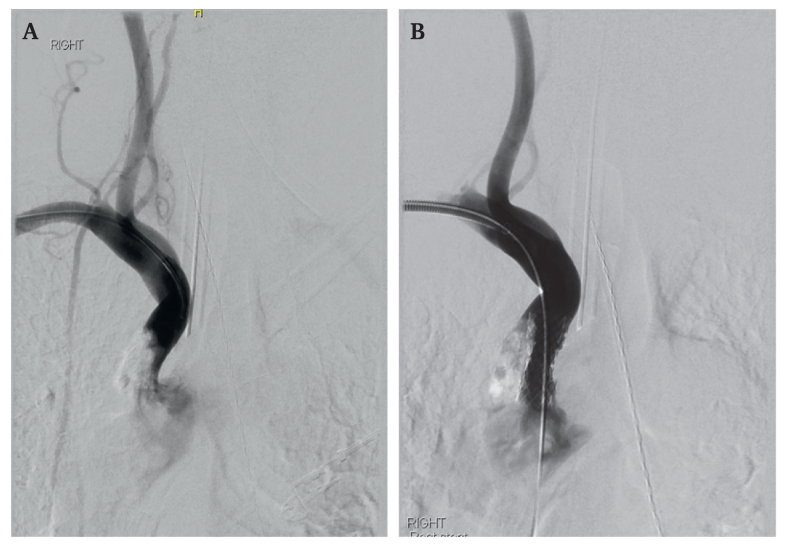
Digital subtraction angiography (A) before insertion and (B) after stent insertion.

Although embolism is the most common mechanism by which extracranial cerebrovascular disease causes cerebral ischaemia, extensive and severe disease can also lead to haemodynamic compromise from hypoperfusion. This is particularly relevant in multivessel disease, where identifying the culprit lesion is challenging, and the risks of peri-operative embolisation and intra-operative hypoperfusion are significant. Haemodynamic stroke, a subtype of ischaemic stroke, arises from reduced cerebral perfusion rather than embolism or small vessel occlusion. It may occur secondary to systemic factors such as heart failure or hypotension, or due to critical stenosis of the carotid or vertebral arteries. Although precise figures vary, hypoperfusion related strokes are implicated in up to 40–50% of cases in certain high risk populations.^1^ Management remains difficult in the absence of randomised trials, with current approaches often guided by case series. Further research is needed to establish diagnostic criteria and explore treatment strategies for this under recognised entity.^2^^,^^3^ (see Figure 1, Figure 2).

## CASE REPORT

A 74 year old woman with severe hypertension and chronic limb threatening ischaemia, presenting with rest pain, was admitted for a femoropopliteal bypass. On induction of general anaesthesia, she experienced a pulseless electrical activity arrest. After a brief period of cardiopulmonary resuscitation, the patient achieved return of spontaneous circulation. Intra-operative transthoracic echocardiography revealed severe left ventricular hypertrophy with dynamic outflow obstruction, but preserved overall cardiac function. The patient remained haemodynamically stable for an extended period following resuscitation. A multidisciplinary discussion with the operating surgeon, anaesthetist, and cardiologist considered the risks and benefits of proceeding. Given the patient's chronic limb threatening ischaemia and stable post-resuscitation status, the team agreed to proceed with the surgery, which was completed successfully.

After surgery, the patient was managed in the intensive care unit. The femoropopliteal bypass remained patent with improved perfusion of the foot. Her pre-operative blood pressure was 165/76 mmHg and cardiology noted a >10 year history of hypertension, with unclear treatment compliance. Transthoracic echocardiography findings were consistent with hypertensive cardiomyopathy, and cardiology recommended strict blood pressure management with a target systolic range of 110–160 mmHg. However, this target appeared poorly tolerated; the patient developed repeated transient ischaemic attacks (TIAs), including left hemiplegia with a blood pressure of <160 mmHg. A code stroke was called with immediate neurology review, and symptoms resolved as the patient was being urgently transported to computed tomography (CT) for stroke protocol imaging. There was no brain infarction on CT imaging, but an increased time to maximum in a small region of the right parietal lobe was identified, with multilevel disease and a sub-occlusive stenosis of the brachiocephalic artery, bilateral carotid bulb stenoses (50–60%), and carotid siphons noted to have bilateral mild to moderate disease with 50% stenoses, and stenoses noted at both right middle cerebral and at intradural vertebral arteries with mild stenosis <50%. All the stenoses appeared chronic with atheromatous plaque and no evidence of thrombus. The circle of Willis was incomplete with a hypoplastic right anterior cerebral artery segment 1 (A1) hampering cross over collateral flow with patent posterior communicating arteries. A second stroke protocol CT performed after recurrence of symptoms again demonstrated hypoperfusion in the right parietal lobe; however, magnetic resonance imaging performed immediately before intervention did not demonstrate restriction of diffusion in this region. Based on CT imaging and carotid duplex evaluation, the brachiocephalic artery was considered the most haemodynamically significant lesion with near complete stenosis of >90%.

Notably, permissive hypertension with a systolic blood pressure (SBP) > 200 mmHg (measured in the left arm) normalised her neurological status, and she was maintained on both anticoagulation and antiplatelet therapy. However, when the SBP fell below 160 mmHg, she experienced symptoms consistent with right hemispheric ischaemia. This phenomenon was observed on four separate occasions during the intensive care unit admission. A diagnosis of recurrent right carotid territory ischaemia secondary to hypoperfusion was made, in the context of intracranial stenosis and systemic hypotension, and imaging findings. Other advanced perfusion imaging modalities were not performed. Following a multidisciplinary meeting with vascular, cardiac surgery, cardiology, neurology, and critical care services, a decision was made to treat the brachiocephalic stenosis with a hybrid approach, with stent placement via the right axillary artery. Open access to provide right common carotid access was considered to allow cross clamping and reduce embolic debris but given the incomplete circle of Willis and the hypoperfusion attacks or TIAs, the risk of stroke from even temporary reduced perfusion was prohibitive. Because the brachiocephalic artery was considered the culprit lesion and the disease was considered hypoperfusive rather than embolic, invasive treatment of the carotid bulb lesion was not considered.

Under general anaesthesia with cerebral oxygenation monitoring using near infrared spectroscopy, the distal right axillary artery was exposed surgically and a direct arteriotomy was performed. After administration of 5 000 international units of heparin an 8 Fr sheath was placed. The brachiocephalic lesion was carefully crossed with a Glidewire (Terumo, Tokyo, Japan) and then exchanged for a stiff wire. A 10 × 29 mm Gore Viabahn VBX Balloon Expandable Endoprosthesis (VBX Stent Graft, Gore Medical, Flagstaff, AZ, USA) was introduced without pre-dilation, positioned slightly proud into the aortic arch and deployed to nominal pressure (10 atm). The stent was not flared to minimise manipulation and risk of emboli. Completion angiography demonstrated a good technical result and no intracranial embolism.

After arteriotomy closure, the patient had a palpable right radial pulse, which was not present before the procedure. The SBP target was reduced and vasopressor support was ceased. The patient recovered without further neurological events and was discharged to a rehabilitation facility three weeks after surgery. Three months after surgery, she was at home with no leg or cerebrovascular symptoms.

## DISCUSSION

This case underscores the complexity of managing cerebral hypoperfusion in patients with multivessel cerebrovascular disease. Identifying the primary culprit vessel was challenging owing to significant disease in multiple locations, including the brachiocephalic artery, carotid bifurcations, and intracranial vessels. Each lesion might have contributed to hypoperfusion, complicating the decision making process for targeted intervention. The choice to stent the brachiocephalic artery was made after careful evaluation of its potential to improve cerebral blood flow while minimising risks such as embolism and re-stenosis. Stenting offered a less invasive option than open surgery, with the added advantage of quicker recovery, but the challenge lay in ensuring adequate procedural safety given the complexity of the lesions. Additionally, single site revascularisation may have only partially mitigated the global hypoperfusion caused by multivessel disease.

Ultimately, the decision to intervene was driven by the patient's recurrent profound ischaemic events when normotensive and the need for very high blood pressure to preserve cerebral perfusion, and after a multidisciplinary meeting with specialists from neurology, neurointerventional radiology, cardiology, intensive care, and vascular surgery.

Open surgical options were also considered for this patient. Aorto-brachiocephalic bypass is recognised as a durable revascularisation approach for extensive brachiocephalic artery disease with reduced risk of intra-operative embolisation;^4^ however, it is invasive, requiring sternotomy and manipulation of the aortic arch, and carries a high risk of peri-operative complications. Mortality rates have been reported up to 10% within 30 days, particularly in patients with significant comorbidities, such as hypertrophic cardiomyopathy and recent haemodynamic instability.^5^ Left to right carotid bypass, though a less invasive open alternative, was not favoured in this patient because of extensive cerebral disease and the need for cross clamping, which carries risks of further ischaemic events and cerebral perfusion instability.

Ultimately, endovascular stenting was chosen owing to its minimally invasive nature and effectiveness in large vessels like the brachiocephalic artery. Although carotid access with cross clamping has been used successfully in similar cases to reduce embolic risk, it was not suitable in this scenario owing to a lack of brain perfusion reserve.

Brachiocephalic artery stenting does offer a less invasive alternative to open procedures; however, the primary concern with stenting is the risk of embolic stroke. Reported stroke rates vary significantly depending on lesion characteristics, patient complexity, and study design. In large cohort studies, stroke risk is reported at 2–5% for isolated brachiocephalic artery stenting in low to moderate risk populations, with higher rates in multilevel cerebrovascular disease cases or heavily calcified vessels.^6^ For example, a multicentre retrospective analysis reported a stroke risk of 3.7%, emphasising the importance of careful patient selection and procedural expertise.^7^ In this case, pre-operative imaging provided critical insights into lesion characteristics, revealing stable atheromatous plaque without thrombus. This finding significantly reduced concerns about embolic risk during the procedure, further supporting the decision to proceed with stenting as a feasible and less invasive option.

Embolic protection devices (EPDs) can reduce peri-procedural stroke risk, with studies showing absolute risk reductions from 6% to 3–4% in high risk groups.^8^ However, their use in brachiocephalic artery stenting is limited. Ostial lesions often preclude conventional femoral access, requiring technically challenging retrograde access via the subclavian or carotid arteries. Flow reversal systems, though effective elsewhere, are impractical for brachiocephalic lesions because of the inability to occlude flow below the stenosis.^8^ In this patient, who experienced ischaemic events even at systolic pressures of 160 mmHg, flow reversal risked exacerbating cerebral hypoperfusion.

In this patient's case, with severe calcified disease, the decision was made not to deploy an EPD given the high complexity of filter placement via brachial access. A review of recent literature underscores the paucity of data for EPD use in large calibre arteries like the brachiocephalic, with most EPD supporting evidence focused on carotid or coronary territories.^8^^,^^9^ This highlights the complexities of risk benefit analysis in multilevel cerebrovascular disease, where the intervention's risks and benefits must be weighed carefully. The decision for single site revascularisation rather than multiple sites was based on the whole team's input and the risks involved with each additional step.

## CONCLUSION

This case highlights the complexity in treating cerebral hypoperfusion caused by brachiocephalic artery stenosis in the setting of multilevel disease. The patient was initially managed medically but owing to recurrent TIAs, she underwent retrograde brachiocephalic artery stenting. This case also underscores the value of multidisciplinary assessment in managing high risk patients where clear solutions are often elusive.

## FUNDING

This research did not receive any specific grant from funding agencies in the public, commercial, or not for profit sectors.

## CONFLICT OF INTEREST

None.
